# The new wave of Congo virus in Pakistan: emerging threat

**DOI:** 10.1186/s41182-023-00559-z

**Published:** 2023-11-14

**Authors:** Isra Masood, Muhammad Junaid Tahir, Ather Naeem, Oadi N. Shrateh, Ali Ahmed

**Affiliations:** 1 Dow International Medical College, Karachi, Pakistan; 2 Shaukat Khanum Memorial Cancer Hospital and Research Centre, Lahore, Pakistan; 3 Punjab Institute of Cardiology, Lahore, Pakistan; 4 Faculty of Medicine, Al-Quds University, Jerusalem, Palestine; 5 School of Pharmacy, Monash University, Bandar Sunway, Malaysia; 6 Ramallah, Palestine

**Keywords:** Congo virus, Pakistan, Crimean–Congo hemorrhagic fever

## Abstract

Congo virus, or Crimean–Congo hemorrhagic fever (CCHF), is a tick-borne disease caused by a single-stranded RNA virus (genus nairovirus, Bunyaviridae family). It spreads through infected ticks' bites or contact with viremic individuals or livestock. Factors supporting its spread include hot, humid climates, limited pesticide use, poor animal control, inadequate irrigation during monsoons, and vector control deficiencies. Nosocomial transmission in under-resourced hospitals poses a threat to healthcare workers. Decades of CCHF cases persist in Pakistan due to these factors, with six deaths reported by June 2023. To combat the epidemic, Pakistan should raise awareness, improve irrigation, establish surveillance systems, and implement livestock quarantine and vaccination.

## Global burden and history

Congo virus, more commonly known as Crimean–Congo hemorrhagic fever (CCHF), was first recognized in 1944 in the Crimean region of what used to be the Soviet Union and then later in 1960 in Congo [1]. CCHF is a negative sense single stranded RNA virus belonging to the genus nairovirus of the Bunyaviridae family [2]. It is a tick-borne illness transmitted to humans directly through the bite of an infected tick or upon contact with blood or tissue of previously viremic individuals or livestock [2]. Since its discovery, CCHF has posed a serious threat for numerous outbreaks on a global scale, especially throughout the regions of Africa, Middle East, and Asia with a case fatality rate of 10–40% [3].

## Burden in Pakistan and history

In particular, Pakistan among other South Asian and Middle Eastern countries has been known for being a yearly target of this vector borne illness. Due to its geographical location, Pakistan’s climate remains a favorable ground for harboring infectious diseases and CCHF is one of its biannual contenders with cases surging each year from March to May and then in July to September [4]. CCHF has been endemic in Pakistan since its initial discovery in Rawalpindi in 1976 after which the number of cases has remained sporadic, surfacing especially around the time of the Islamic holiday of *Eid al Adha* [1]. The first ever CCHF case in Pakistan from Rawalpindi was seen at the city’s General Hospital and resulted in 11 secondary cases and 3 deaths making it one of the first ever reported high risk nosocomial infections in Pakistan [4]. Shortly thereafter, the disease made its way through to other provinces in the country with cases mostly emerging now from Balochistan and Sindh. The current year’s statistics for Pakistan have reported a total of 6 deaths at the hands of CCHF till June of 2023 with potential for more [5]. Like every year, if the appropriate precaution at the appropriate time is still not taken, these reports could be warning signs towards a new wave of the CCHF endemic in Pakistan.

## Clinical presentation and management

Within the first few days of contact, CCHF can induce critical damage to the infected individual leading to many serious and possibly fatal outcomes. The common initial symptoms of this illness present as fever, headaches, myalgia, fatigue, and gastrointestinal distress. In severe cases, the manifestations may progress to hemorrhages including gingival and petechial hemorrhages, epistaxis, ecchymoses, and other forms of extensive bleeding [2]. Currently, the available diagnostic tests for CCHF include a reverse transcription–polymerase chain reaction (RT–PCR) assay, enzyme linked immunosorbent assay (ELISA), detection of antigen-specific antibodies, or through isolating the virus on cell culture [6]. The primary treatment for CCHF is supportive care and management of symptoms, however in some cases oral and intravenous administration of ribavirin, an antiviral drug, has also shown benefit [6]. Two vaccines against the virus have also been developed but neither of them has been approved for administration as of yet [1].

## Risk of emergence

According to the statistics provided by the national institute of health (NIH), there were a total of 365 reported CCHF cases and a 25% fatality rate in Pakistan between the year of 2014–2020 [1]. In 2021, 14 confirmed cases and 6 fatalities were reported in the province of Balochistan [4]. Then in 2022, there were 4 reported cases in the first 5 months of the year followed by a surge of 7 more cases from Balochistan and Khyber Pakhtunkhwa in the second half of the year [4]. As of June 3rd 2023, a total of 6 deaths have been reported in the country [5]. One of the reported fatalities was of a 28-year-old meat seller from Karachi who initially developed a fever on April 30th 2023 shortly after which his health deteriorated [7]. The individual had initially been tested for dengue and malaria, both of which tests came out to be negative. This entails that perhaps the delay in treatment due to confusion of which virus infected the individual could have possibly led to the patient’s untimely demise. This pattern of yearly CCHF statistics in Pakistan can be highly suggestive of possible spread of infection in livestock that is brought to the city from rural regions especially around the time of Eid al Adha. The animals brought for sacrifice to the bigger cities come from rural regions where vector control practices are not implemented leading to a higher risk of transmission of disease from the ticks to the livestock [4]. The infected livestock is then butchered without the use of gloves or the appropriate sanitary methods and no prior quarantining or vaccination of the animals. Direct contact with blood and tissue of the diseased animal can then lead to CCHF infection in previously healthy individuals with the risk of spreading to other individuals through the same method of transmission and so on.

## Seasonal distribution

In recent years, the heavy monsoon seasons in Pakistan have also raised suspicions regarding the surge in CCHF cases. Lack of proper irrigation systems throughout the country create vast bodies of still water on the streets that become the perfect environment for more infectious diseases to spawn including dengue, malaria, and cholera [4]. This addition to the already devastating aftereffects of the floods makes the situation more debilitating especially for a third world country like Pakistan. The constant political unrest in the country has also made it so that no financial aid or attention for that matter can be given to assist in fixing the situation, rendering the health district authorities helpless in combating the recurrence of these detrimental diseases. In 2022, the UN and Pakistan had reportedly asked for $160 million to provide shelter, food, and sanitation to 5 million people rendered homeless because of the floods highlighting the severity of debt and poverty in the country [8].

## Current actions taken and need of the hour

The Sindh Government of Pakistan advises the District Health Officers (DHOs) to implement standard operating procedures (SOPs) prior to the holiday of Eid al Acha each year. The SOPs include wearing gloves during the sacrificial process of the animal to strictly avoid contact with the animal’s blood or other bodily fluids in an attempt to limit the spread of the virus and prevent fatalities [9]. In recent news, the government of province Khyber Pakhtunkhwa has also begun to take strict measures establishing checkpoints at its borders for animal crossing to ensure all livestock brought past the borders has been disinfected with anti-tick spray [10]. Despite the efforts however, there is still a lack of quarantining of animals and their vaccination against CCHF as well as the lack of equal implementation of these practices in all provinces of the country. Appropriate vector control measures also remain absent in the rural regions where most of the cases emerge.

## Health education

Being an underdeveloped country, Pakistan particularly does not possess adequate equipment nor the financial means necessary for establishing a sustainable and efficient healthcare system that is crucial in endemics or other health crises. Raising awareness through campaigns and other public awareness techniques such as influential media channels to educate people is a vital precautionary measure that should be taken to prevent future CCHF endemics. The veterinary sector should educate people on vectors that carry risk of transmission to animals and humans such as ticks that are amplifying hosts for many diseases including CCHF.

There is also a very prominent lack of education and training amongst healthcare workers on handling emergency situations and taking the appropriate precautions when necessary. The curriculum for healthcare staff should be reevaluated to incorporate CCHF specific guidelines and regulations that address the clinical presentation and manifestations of the illness in detail as well as the required lab tests and treatment. In doing so, diagnostic approaches can also be revised in order to differentiate the clinical presentation of CCHF with that of other infectious illnesses that are more prevalent in Pakistan such as malaria and dengue virus.

The government of Pakistan should also think it necessary to provide the healthcare industry with sufficient funds for CCHF specific testing services and institutes to help in early detection and screening of the disease. In public health crises like these, awareness and contributions from the community tend to make the most positive impact so ensuring that everyone in the community does their part is also a vital component of reform in this regard. Pushing for investments towards an efficient irrigation system throughout the country would also prove to be fruitful for future monsoon seasons. Limiting crossing the border with cattle from other CCHF endemic regions especially during the time of the year when cases tend to rise could also show benefit in keeping the virus from spreading. For animals sold during Eid-ul-Adha, strict guidelines that ensure pesticide use and vaccination of the livestock should be issued so the virus can stay confined to one area making it easier to eradicate it [4]. In addition, livestock from endemic regions should be quarantined prior to being brought to public markets.

## Conclusion

As for the rural community where the majority of the cases emerge, regular screenings of the civilians should be made an annual or biannual practice. Lastly, the health district should also establish a surveillance system or local database to keep official records of cases and other CCHF related statistics for early detection and prevention of potential future outbreaks. All undergraduate programs offering courses in community medicine and health should accommodate sentinel surveillance as part of the curriculum in order to highlight its importance and its benefits to the health community regarding such endemic diseases.

## Data Availability

The data used to support the findings of this study are available from the corresponding author upon reasonable request.

## Abbreviations

CCHF: Crimean–Congo hemorrhagic fever
RT–PCR: Reverse transcription–polymerase chain reaction assay
ELISA: Enzyme linked immunosorbent assay
DHOs: District Health Officers
SOPs: Standard operating procedures
